# Exploring academic achievement and relevant risk factors among a community sample of adolescents with chronic pain compared to peers

**DOI:** 10.1093/jpepsy/jsaf015

**Published:** 2025-04-12

**Authors:** Darragh Mullen, Melissa Pielech, Agnieszka Graham, Anthea Percy

**Affiliations:** School of Psychology, Queen’s University Belfast, Belfast, United Kingdom; Department of Psychiatry and Human Behavior, Warren Alpert Medical School of Brown University, Providence, RI, United States; Department of Behavioral and Social Sciences, Center for Alcohol and Addiction Studies, Brown University School of Public Health, Providence, RI, United States; School of Psychology, Queen’s University Belfast, Belfast, United Kingdom; School of Psychology, Queen’s University Belfast, Belfast, United Kingdom

**Keywords:** chronic pain, pain, adolescents, ALSPAC, school, academic achievement, substance use, bullying, sleep, mental health, depression

## Abstract

**Objective:**

To compare adolescents in the United Kingdom with chronic pain with their peers in relation to psychological and behavioral outcomes (i.e., mental health, bullying, substance use) and academic achievement.

**Methods:**

Participants were adolescents with chronic pain (*n* = 856) and peers without chronic pain (*n* = 3,093) from the Avon Longitudinal Study of Parents and Children (ALSPAC) who attended a research clinic in the United Kingdom at 17 years and completed data collection at multiple timepoints. Chi-square and *t*-tests were used to explore group differences across psychological and behavioral measures. Regression and mediation analyses examined the relationship between chronic pain and academic achievement measures, including the derived variables of pathway to higher education and educational qualifications.

**Results:**

Adolescents with chronic pain were found to experience more difficulties with mental health, bullying, and substance use. Additionally, a relationship between chronic pain and reporting a pathway to higher education was found after key variables were accounted for, although group differences were not observed across other academic achievement measures. Further analyses identified a moderate indirect effect of chronic pain on reporting a pathway to higher education when mediated by sleep difficulties.

**Conclusions:**

The limited predictive relationship between chronic pain and academic achievement potentially indicates that, despite struggling more with factors such as mental health, bullying, and substance use, adolescents with chronic pain may utilize enhanced skills in maintaining a developmental trajectory at school or external factors such as support from their caregivers or school. The complex interrelationship between sleep and chronic pain is also an important consideration for the ability to achieve academically.

Chronic pain is a common condition in adolescence which can impact daily life, including the school experience and other psychological and behavioral outcomes. Chronic pain is defined by the International Association for the Study of Pain (Merskey & Bogduk, 1994) as pain which lasts for more than 3 months, with the International Classification of Diseases distinguishing between “primary” and “secondary” forms of chronic pain (ICD-11; Treede et al., 2015). Reported prevalence rates among youth range from between 26%–69% for chronic headaches and 8%–53% for chronic abdominal pain (King et al., 2011), with higher prevalence among females, older adolescents, and those of lower socioeconomic status (SES; Wager et al., 2020).

Chronic pain has the potential to negatively impact psychosocial functioning and development, including a greater risk of peer victimization (Fales et al., 2018). Mental health comorbidities are also common among young people with chronic pain, with higher rates of depression and anxiety frequently reported compared to their peers (Wager et al., 2020). Although experiencing persistent pain has been hypothesized as a risk factor for substance use (Ditre et al., 2019), rates of substance use among youth with chronic pain are not well understood. In one sample, increased levels of alcohol consumption, smoking, and illicit drug use were observed among adolescents whose chronic pain was described as “problematic” (McLaren et al., 2017), yet another sample identified lower rates of substance use among youth with chronic pain compared to peers (Law et al., 2015). Psychosocial difficulties and functional disability have been linked to chronic pain-related sleep difficulties (Heyer et al., 2014), whereby a complex, bidirectional relationship between sleep and persistent pain can heighten the risk of developing chronic pain, increase daily fluctuations of pain intensity, and worsen the long-term prognosis for some chronic pain conditions (Finan et al., 2013). To date, however, limited research has explored the impact of such sleep difficulties on school functioning in a community-based population of young people with chronic pain.

Chronic pain frequently impacts school attendance as well as the ability to keep up with schoolwork, make friends, and develop educational and social skills (Logan et al., 2009). This in turn can contribute to a negative cycle of nonattendance and lower self-esteem that is maintained by underdeveloped social and academic skills (Vitulano, 2003). The ability to achieve in school is dependent on a variety of interrelated personal, psychosocial, and contextual factors (Vermunt, 2005). As such, academic achievement is a useful proxy measure of overall school functioning, whereby direct measures of academic achievement provide an objective measure that is relatively stable over time (Bacon & Bean, 2006), standardized nationally (Modin et al., 2015), and is a strong predictor of an individual’s enjoyment of school generally (Morris et al., 2021).

Mixed findings have been observed regarding relations between different chronic pain conditions and academic achievement (Devanarayana et al., 2008), although there appears to be evidence that a negative impact is frequently present (Turk & Şahin, 2020). It is noted across the literature, however, that studies do not tend to account for some of the range of confounding factors which may affect this relationship, such as SES, IQ, or parental education (Bergman & Joye, 2001), and no such studies appear to have been undertaken with a U.K. population. Similarly, despite known influences for both chronic pain and academic achievement, such as sleep, fatigue, or concentration (Clementi et al., 2020; Solberg Nes et al., 2009), there appears to be a dearth of research exploring their mediating or moderating effects. Furthermore, it is contextually important to explore the prevalence and severity of psychosocial factors which relate to academic achievement among community chronic pain populations (which includes adolescents who may or may not be receiving pediatric pain care from medical professionals), and to compare this with a community sample without chronic pain to explore normative trends and place frequency rates in context.

Through a secondary analysis of an epidemiological community-based cohort from the United Kingdom, the current study aimed:

Aim 1: To compare academic achievement, mental health, health behaviors, and social factors among adolescents with chronic pain versus their peers without chronic pain at age 17 years.

Aim 2: To examine whether group differences in academic achievement between adolescents with chronic pain and their peers without chronic pain exist after controlling for key sociodemographic factors including sex, IQ, SES, and parental education.

Aim 3a. To assess how specific pain characteristics for the group of adolescents with chronic pain may relate to academic achievement measures.

Aim 3b: To examine the extent to which relations between chronic pain and academic achievement may be mediated by problems with sleep, fatigue, and concentration, which are common secondary outcomes of chronic pain and which are also known to impact academic achievement. The potential moderating effect of specific pain characteristics on these relationships will also be explored.

## Methods

### Study sample

Data for the current study were extracted from the Avon Longitudinal Study of Parents and Children (ALSPAC). Study aims and analyses were established and submitted for review prior to being granted access to the data. ALSPAC is a cohort of children born between April 1991 and December 1992 in the County of Avon in the United Kingdom (Boyd et al., 2013; Fraser et al., 2013; Northstone et al., 2019) which assessed a wide range of factors relating to the lives, health, development, and psychosocial development of this group.^1^ The study sample included adolescents who completed a questionnaire about pain at ∼17 years of age (*N* = 4,000). Other variables were collected for this group at earlier and later data collection points across the longitudinal study, including descriptive, outcome, mediating, and control variables.

#### Chronic pain-related measures

##### Pain information

At age 17 years, youth in ALSPAC completed a pain questionnaire, adapted from a robust, validated measure previously used with youth in the United Kingdom which included information on pain history, pain locations, when pain was most troublesome, and days it kept participants from doing activities (Mallen et al., 2006). Participants were initially asked if they had experienced any aches or pains for a day or longer in the previous month. If they responded “yes,” participants were asked if the pain had begun “less than” or “more than” 3 months prior, with chronic pain operationalized as pain that persists for longer than 3 months (Merskey & Bogduk, 1994). Adolescents who reported experiencing pain for longer than 3 months were assigned to the chronic pain group, while those who reported no pain or pain that had not persisted for at least 3 months were assigned to the comparison peer group, which reflected the typical, everyday pain experience of those who experienced no pain in that period or pain which was not chronic.

##### Pain intensity and disability

The pain questionnaire, completed at age 17 years, also assessed the intensity of the worst pain and the average intensity of the worst pain in the previous 6 months (Mallen et al., 2006). Pain interference was assessed by asking about the impact that pain had on daily activities on a scale of 1 (no interference) through 10 (unable to carry out activities), and any change in the ability to take part in daily activities (1 = no change, 10 = extreme change). As per Fisher et al. (2016), the two scores were combined to calculate a mean score which reflected pain-related disability. Internal consistency in the current sample was good (α = .89).

##### Pain-specific anxiety

This was assessed at age 17 years via a 7-item subscale of the Bath Adolescent Pain Questionnaire (BAPQ) which has been validated with a tertiary chronic pain sample and a rheumatology outpatient sample (Eccleston et al., 2005). Adolescents reported how often they experienced these worries on a 5-point Likert-type scale (from “Never” = 0 to “Always” = 4), with a total score computed from these seven items (range 0–28), with higher anxiety indicated by higher scores. Internal consistency of the current sample was good (α = .78).

#### Academic achievement measures

Academic achievement measures were obtained via self-report at ages 18 and 20 years. Participants reported the qualifications (i.e., examinations) and degrees they obtained from the General Certificate of Secondary Education (GCSE; usually taken at age 16 years; roughly similar to a high school diploma or equivalent in the United States), to A-levels (taken between the ages of 16–18 years for the purpose of attending university), and onward. Details of each academic achievement measure and equivalent measures in the United States, when applicable, are included in Table 1.

**Table 1.** Academic achievement and mental health outcomes for adolescents with chronic pain (CP) and their peers without chronic pain (NCP) in the United Kingdom.

**Academic Achievement** ^†^	CP (*n* = 856)	NCP (*n* = 3,093)	**χ^2^** (2)	p*
**General Certificate of Secondary Education (GCSE) Grade** GCSE are main qualifications taken at 14–16 years, but available to anyone of any age. While there is no direct equivalent of the GCSE in the United States, the requirements of a high school diploma or General Education Development (GED) are the most similar. GCSE is scored on a 9-point scale. The highest grade is an A* and the lowest grade is G (akin to scores of A+ and F in the United States). Scores of C and above are considered passing. Successfully passing the GCSE is a prerequisite to taking A-Level exams. In the current dataset, GSCE variables were self-reported by adolescents at age 18 years.				
Obtained a score of A*–C on the GCSE (*N* = 2,061)	50.7	50.8	.001	>1.0
*Obtained a score of D–G on the GCSE* (*N* = 1,903)	19.5	18.6	1.24	>1.0
**A-Level or “Advanced Level” Qualifications** Advanced Subsidiary (AS) level and Advanced (A) level qualifications exams are taken after the GCSE, split into two parts, and typically take 2 years to complete. The first part is known as the AS-level, which is also a qualification in its own right. Part two is known as the A2 level. The complete A Level qualification is formed by combining the AS and A2 Levels. The highest grade is an A* (akin to A+ in the United States) and the lowest grade is U (Unclassified). A passing grade corresponds to an E or above (>40%). The data used in this study did not include information on the number of A-Levels a participant completed or the specific grades received. In the United States, the closest equivalent to A/AS Levels are Advanced Placement (AP) exams because both systems provide students with an opportunity to study advanced subject matter. In the current dataset, A-Level variables were assessed at age 18 years.				
*Obtained A/AS Levels* (*N* = 1,989)	40.5	41.4	1.54	>1.0
*Obtained A/A2 Levels* (*N* = 1,936)	28.7	32.2	6.38	.13
**Applying to University** *Students in the United Kingdom typically apply to University when they are age 18 years. The most common pathway for students applying to university is completing A-levels, although other entry routes are in place through other qualifications or diplomas equivalent to A-Levels. Similar to the admissions process in the United States, U.K. universities vary in the number of subjects they require, which subjects are required, and the specific grades required at a minimum. Among the variables reported here, participants would need to meet eligibility criteria to apply for university, such as by completing A-levels. Being awarded a place reflects that a young person met the acceptance criteria for their course, such as by achieving the appropriate grades, which students apply to in order of preference. In the current dataset, variables related to applying to university were self-reported at age 18 years.*				
Applied to University (*N* = 2,071)	30.7	32.8	1.81	>1.0
Awarded University Place (*N* = 1,261)	22.2	25.3	0.94	>1.0
Received First University Choice (*N* = 966)	17.9	20.6	0.21	>1.0
**Degree-level qualifications** *Attainment of degree-level qualifications (e.g., BA, BS) was assessed at age 20 years* *A degree-level qualification refers to those achieved at higher education, such as a Bachelor’s Degree. It is noted that participants in higher education at age 20 years may not yet have graduated and obtained a degree by the time the data were recorded.* *HNC is a level-4 vocational qualification that takes 1 year to complete and which enables individuals to prepare for work or studies at a degree level. HND is a level-5 qualification which takes 2 years to complete, preparing individuals for work or study toward a Bachelor’s Degree, similar to an Associate’s Degree of the first 2 years of a Bachelor’s Degree program in the United States.* *ONC and OND are level-3 further education qualifications in the United Kingdom and are broadly comparable with A-levels*				
Has degree-level qualification (*N* = 2,274)	15.1	13.9	1.99	>1.0
Has HNC/HND qualification (*N* = 2,190)	1.2	1.3	.05	>1.0
Has ONC/OND qualification (*N* = 2,177)	1.4	0.7	4.33	.41
**Pathway to Higher Education** *A dichotomous variable (yes or no), pathway to higher education, was operationalized based on self-report of attending higher education and/or achieving school grades or a degree which would enable the participant to meet the admission criteria of higher education programs. Participants were deemed to be, or not to be, on a pathway to higher education based on their responses to 22 questions, e.g., “Young person plans to leave full time education and do something else”*=no, “Respondent obtained qualification: GCSE grades A*”=yes*, “Respondent awarded place at university”* *=* *yes. If a participant responded affirmatively to any question related to pursuing higher education, they were placed to be in the pathway to higher education group.*				
**On a pathway to higher education** **(*N* = 2,833)**	78.6	80.8	1.45	.229
**Educational Qualifications** A continuous variable, educational qualifications, was computed reflecting the amount and difficulty of qualification(s) or degree(s) a participant obtained as they progressed through education, e.g., “Obtained GCSE grade A*–*C = 1 point,” “Obtained A/A2s levels = 2 points,” etc., with higher scores indicating obtaining advanced qualifications of increasing difficulty. These were summed to create a total score based on the number of qualifications reported by a participant, with a theoretical maximum score of 17, although such a score is not expected because it is unlikely that someone would obtain all possible degrees.*				
**Educational qualifications score**	*M* = 4.06	*M* = 4.13	*t* (2,810)=0.48	
**(*N* = 2,810)**	*SD*=3.23	*SD* = 3.18		0.92

**Mental Health Diagnoses**	CP	NCP	**χ^2^** (2)	p*
	(*n* = 771)	(*n* = 2,761)	(*N* = 3,532)	
General Anxiety Disorder	10.2%	4.5%	37.76	.01
Panic Disorder	1.9%	0.7%	11.10	.05
Social Phobia	2.9%	1.8%	3.22	>1.0
Specific Phobias	6.1%	2.8%	20.28	.01
Chronic Fatigue Indicator	13.1%	6.5%	35.93	.01
Major Depressive Disorder (as defined by ICD-10 Depressive Criteria	14.7%	6.3%	56.00	.01

**Depressive Symptomology Relevant to Academic Functioning** ^‡^	CP	NCP	**χ^2^** (2)	p*
	(*n* = 768)	(*n* = 2,753)	(*N* = 3,521)	
Significant Sleep Difficulties	31.3%	21.9%	29.06	.01
Significant Concentration Difficulties	17.3%	9.1%	42.02	.01
Significant Fatigue Difficulties	42.4%	29.1%	49.19	.01
Significant Worry Difficulties	27.9%	19.5%	25.00	.01

**Directly Bullied in last 6 months**	CP	NCP	**χ^2^**(3)	*p* *
	(*n* = 700)	(*n* = 2,476)	(*N* = 3,176)	
Never	87.7%	91.2%	21.71	.01
“Not much” (1–3 times)	10.1%	8.2%		
“Quite a lot” or “A lot” (>4 times)	2.2%	0.6%		

**Relationally Bullied in last 6 months**	CP	NCP	**χ^2^**(3)	*p* *
	(*n* = 700)	(*n* = 2,470)	(*N* = 3,170)	
Never	73.7%	80.7%	20.02	.01
“Not much” (1–3 times)	22.1%	17.2%		
“Quite a lot” or “A lot” (>4 times)	4.1%	2.1%		

**Smoking**	CP	NCP	**χ^2^** (1)	p*
Ever smoked a whole cigarette (*N* = 3,259)	56.1%	48.9%	11.623	.004
Has smoked in past 30 days (*N* = 1,643)	31.7%	26.2%	0.960	>1.0
Smokes every day (*N* = 894)	16.3%	10.6%	8.678	.01
Smokes every week (*N* = 894)	20.1%	15.1%	4.923	.10

**Alcohol Use**	CP	NCP	**χ^2^** (1)	p*
Ever drunk a whole alcoholic drink (*N* = 3,257)	96.0%	94.3%	3.06	.80
Mean age of first alcoholic drink (*N* = 3,074)	*M* = 13.59	*M* = 13.84		
	*SD* = 1.96	*SD* = 1.84		

**Cannabis Use**	CP	NCP	**χ^2^** (1)	p*
Ever tried cannabis (*N* = 3,224)	44.4%	38.9%	3.06	.03
Mean age of first cannabis use (*N* = 1,288)	*M* = 15.00	*M* = 15.20		.
	*SD* = 1.49	*SD* = 1.57		

*Note*.

* Adjusted *p*-values using Bonferroni corrections.

† Variance in *N* for academic achievement measures due to missing responses.

‡ Measured during completion of CIS-R. Score of 2 or greater deemed clinically significant.

Two composite variables representing academic achievement (“pathway to higher education” and “educational qualifications”) were created, which are described in detail in Table 1. “Pathway to higher education” is a dichotomous variable operationalized based on self-report of attending higher education and/or achieving school grades or a degree which would enable the participant to meet the admission criteria of higher education programs. “Educational qualifications” is a continuous score reflecting the amount and difficulty of qualification(s) or degree(s) a participant obtained as they progressed through education, e.g., “Obtained GCSE grade A*-C = 1 point,” “Obtained A/A2s levels = 2 points,” etc, with higher scores indicating obtaining advanced qualifications of increasing difficulty. For this variable, the maximum score (17) was not expected because it is unlikely that someone would obtain all possible degrees. At later data collection points, there were more missing data related to academic achievement because fewer students achieved the most advanced degrees and some students had not completed their degree by the point the data were collected (age 20 years). Thus, these composite academic achievement variables sought to optimize the utilization of the information available. The list of questions used to compute the variables is included in the Supplementary Material.

#### Mental health-related measures

##### Mental health diagnoses, depressive symptoms, sleep, concentration, and fatigue

These were assessed via self-administered computerized interview schedule (CIS-R; Lewis et al., 1992) at the same time as the pain questionnaire was completed (age 17 years). The CIS-R was designed to measure the severity and nature of mental health-related symptoms and diagnoses (i.e., anxiety, phobias, depressive episodes), based on ICD10-F32 criteria. The CIS-R has been validated in a community setting with a population aged 16–64 years (Brugha et al., 1999). Difficulties with sleep, concentration, and fatigue were assessed via depressive symptoms subscales from the CIS-R. Each subscale produced a score of 0–4, with a score of 2 or greater indicating a clinically significant difficulty in this area.

#### Health and social behavior measures

##### Substance use and peer victimization

Onset and frequency of substance use were self-reported at 17 years using the Alcohol Use Disorders Identification Test (AUDIT; 10 items and total score of 0–40; Reinert & Allen, 2002), the Fagerström Test for Nicotine Dependence (FTND; six items and total score of 0–10; Heatherton et al., 1991) and the Cannabis Abuse Screening Test (CAST; 4 items and total score of 0–6; Legleye et al., 2007). Scores were categorized based on risk categories for each measure. Bullying was self-reported at 17 years, with participants reporting the number of incidents of being directly or relationally bullied in the previous 6 months.

#### Sociodemographic characteristics

Sex and ethnicity data were recorded at birth, with terms used for ethnicity as they were collected in the original survey. Parental education and social class from parental occupation were measured at 32 weeks gestation, as was SES, which was recorded using a Cambridge Social Stratification Score (CAMSIS) which aims to provide a more flexible measure of SES through accounting for social heterogeneity (Bergman & Joye, 2001). The highest level for both measures was calculated using the dominance model based on responses by the mother or her partner (Erikson, 1984).

#### IQ

This was measured during a study visit at age 8 years via completion of a short form of the Wechsler Intelligence Scale for Children (WISC-III^UK^), which is a reliable and valid measure of cognitive ability (Wechsler, 1991). Full-scale IQ score (combining verbal and performance IQ subscale scores) is reported for this study.

### Data analyses

All analyses in this study were undertaken using SPSS version 28.0. After data cleaning, descriptive statistics and frequency analyses were calculated for sociodemographic information and relevant psychosocial factors of mental health, rates of substance use, and peer victimization, with Bonferroni corrections applied to *p*-values when multiple comparisons were made. Analyses for Aim 1 investigated group differences by conducting *t*-tests and chi-square tests across academic achievement measures and other variables related to mental health, health behaviors, and social factors. Pearson correlation and point-biserial coefficients were undertaken to ensure no variables in subsequent models were highly correlated or measuring the same construct. This was followed by logistic and linear regression models which explored the extent to which chronic pain predicted academic achievement measures after confounding variables of sex, IQ, SES, and parental education were accounted for (Aim 2). As per Aim 3a, *t*-tests were conducted within the chronic pain group to explore how the specific pain characteristics of pain intensity, anxiety, and disability may relate to measures of academic achievement. For Aim 3b, combined regression and mediation analyses were performed using Hayes’ PROCESS Macro plugin software for SPSS (Hayes, 2022), which explored sleep, concentration, and fatigue difficulties as potential mediating factors in the relationship between chronic pain and academic achievement. These were first examined as single mediators before exploring combinations of mediators sequentially. The moderating effect of pain characteristics on this relationship was also explored. As per Preacher and Hayes (2004), 95% confidence intervals were derived from bootstrapping test with 5,000 resamples, with a significant mediation effect occurring if confidence intervals excluded zero.

## Results

### Participants

A total of 4,000 participants completed the pain questionnaire at ∼17 years of age. Of this total sample, 1,828 adolescents reported experiencing pain that lasted for a day or longer in the previous month, while 2,124 adolescents reported that they had not. Recent pain was reported by 969 adolescents, however, this did not meet the criteria of chronic pain, i.e., pain which persisted for more than 3 months. Of the 1,828 adolescents who reported pain, 856 participants indicated that their pain was chronic. The comparison peer group, which was assumed to represent the “typical” experience of reporting no pain in that period or pain that was not chronic, had a sample size of 3,093.

Sociodemographic and pain-related characteristics for the chronic pain group and their peers without chronic pain are presented in Table 2. A higher proportion of the chronic pain group was female compared to their peers without chronic pain, although this association was weak (χ^2^[1, *N*  =  3,947] = 24.60, *p*<.001, *Cramer’s V* = .08).

**Table 2.** Descriptive information for CP and NCP groups.

	CP (*n* = 856)	NCP (*n* = 3,093)
Variable	%	%
Sex		
Female	65.8	56.4
Male	34.1	43.6
Adolescent’s Ethnic Group		
White	84.5	85.3
Non-White	4.1	4.1
Missing/Not Recorded	11.4	10.6
School Type		
State School	60.0	60.9
Private School	10.4	10.1
Other	1.6	0.9
Not Completed/Missing	28.0	28.1
Highest Parental Education Level		
CSE	7.1	6.8
Vocational	4.4	4.1
O-Level	19.3	20.9
A-Level	32.5	31.5
Degree	26.3	27.8
Not Completed/Missing	10.4	9.0
Highest Social Class from Parental Occupation		
I—Professional	2.5	2.9
II—Managerial and Technical	19.9	22.5
III NM—Skilled Nonmanual	23.4	21.8
III M—Skilled Manual	24.5	25.5
IV—Partly Skilled	9.8	8.5
V—Unskilled	2.5	2.4
Not Completed/Missing	17.7	16.4
**Pain Characteristics**		
When was pain most troublesome in past 6 months		
No score/No response	2.6	69.3
0–7 days	28.4	14.7
1–4 weeks	22.9	9.6
1–3 months	17.9	5.1
>3 months	28.3	1.2
Number of days pain kept from doing usual activities		
0–6	80.1	n/a
7–14	9.1	
15–30	4.8	
31+	4.4	
Pain area >3 months		
Knee	11.2	n/a
Hip	4.0	
Shoulder	10.4	
Lower back	13.7	
Chronic regional pain	23.6	
Chronic widespread pain	21.1	
Intensity of worst pain in past 6 months	*M* = 7.1	n/a
(1–10)	*SD* = 2.2	
Average intensity of worst pain in past 6 months	*M* = 4.9	n/a
(1–10)	*SD* = 2.2	
Pain Anxiety Score	*M* = 12.9	n/a
(0–28)	*SD* = 5.2	
How much pain interfered with usual daily activities (1–10)	*M* = 3.7 *SD* = 2.6	n/a
How much pain has changed ability to take part in recreational, social, and family activities (1–10)	*M* = 3.0 *SD* = 2.4	n/a
		

### Aim 1. Differences in academic achievement, mental health, health behaviors, and social factors between adolescents with chronic pain and their peers without chronic pain

Responses to self-reported measures of school grades and qualifications in each group are outlined in Table 1. No group differences were found across any of the included measures of academic achievement or in the derived variables of pathway to higher education or educational qualifications.

Table 1 also describes psychosocial factors in the chronic pain group and their peer group without chronic pain. Adolescents with chronic pain more frequently met the criteria for a range of mental health diagnoses. Additionally, adolescents with chronic pain reported more clinically significant difficulty scores for sleep, concentration, fatigue, worry, and anxiety, and more frequently reported being directly bullied and relationally bullied. Across analyses of substance use between the two groups, adolescents with chronic pain were more likely to report ever smoking a cigarette and smoking every day. Adolescents with chronic pain also reported first drinking at a younger age than their peers (*t*[3,074] = 3.073, *p* = .006, *d* = .13), were more likely to report ever trying cannabis, and more frequently produced scores that indicated a higher risk level of cannabis misuse compared to peers (chronic pain_*M*_ = 0.41, chronic pain_*SD*_ = 1.11, peers_*M*_ = 0.26, peers_*SD*_ = 0.86; *t*[876] = 2.06, *p*<.001, *d* = .16), although effect sizes were small for both.

### Aim 2. Regression of chronic pain on academic achievement measures

*Do group differences in academic achievement between adolescents with chronic pain and their peers without pain persist after controlling for key sociodemographic factors*?

Correlation analyses were first undertaken to explore associations between variables (see Table 3), which indicated that collinearity was not a concern for subsequent analyses. As outlined in Table 4, a hierarchical logistic regression was undertaken which sequentially introduced the identified covariates of sex, IQ, SES, and parental education as predictor variables in model 1 (χ^2^[7, *n*  =  2,212] = 339.68, *p*<.001) before introducing chronic pain in addition to these in model 2 (χ^2^[8, *n*  =  2,212] = 344.41, *p*<.001) to explore any variance in the outcome variable of pathway to higher education. This found that chronic pain was a significant predictor of pursuing a pathway to higher education after covariates were accounted for, with a reduction in deviance observed in the −2 log-likelihood value for the second model and the significance value in the Hosmer and Lemeshow test for both models indicating an overall good fit.

**Table 3.** Intercorrelations for combined CP and NCP groups between variables in regression and mediation models.

	1	2	3	4	5	6	7	8	9	10	11	12	13
1. Chronic Pain^†^	–	−.02	−.01	.11**	.12**	.14**	.57**	.18**	.30**	.08**	.00	−.01	−.02
2. Pathway to Higher Ed.^†^		–	.34**	−.08**	−.03	−.01	.08**	−.08*	−.05*	.02	.33**	.17**	.12**
3. Educational Qualifications			–	−.04	−.00	−.02	−.05*	−.08*	−.04	.07**	.23**	.09**	.08**
4. Sleep				–	.31**	.39**	.20**	.12**	.18**	.10**	−.03	−.04	−.03
5. Concentration					–	.45**	.19**	.14**	.22**	.08**	.01	−.01	.01
6. Fatigue						–	.25**	.16**	.24**	.20**	.01	−.02	.01
7. Pain Intensity							–	.45**	.42**	.14**	−.07**	−.04	−.02
8. Pain Disability								–	.45**	.12**	−.08*	−.04	−.02
9. Pain Anxiety									–	.14**	−.07**	−.05*	−.05*
10. Sex^†^										–	−.06**	−.02	−.02
11. IQ											–	.20**	.16**
12. SES												–	.71**
13. Parental Education													–

*Note.* Sleep, concentration, and fatigue difficulties measured as depressive symptomology during CIS-R; Pain disability measured from combined questions of reported pain interference and impact on daily activities; Pain anxiety measured using BAPQ; SES measured using CAMSIS scores.

* *p*<.01.

** *p*<.001.

† Dichotomous variables were investigated using point-biserial correlation coefficients: Chronic Pain=yes/no; Pathway to Higher Ed.=yes/no; Sex=male/female.

**Table 4.** Hierarchical binary logistic regression models for pathway to higher education.

	*Model 1*							*Model 2*						
	*B*	*SE*	*Wald* χ^2^	*p*	*OR*	*95% CI*		*B*	*SE*	*Wald* χ^2^	*p*	*OR*	*95% CI*	
Sex^†^	.30	.12	5.93	.02	1.35	1.06	1.72	.33	.13	6.99	.01	1.39	1.09	1.78
IQ	.05	.00	119.85	<.001	1.05	1.04	1.06	.05	.00	121.04	<.001	1.05	1.04	1.06
SES	.03	.01	27.23	<.001	1.03	1.02	1.05	.03	.01	27.44	<.001	1.03	1.02	1.05
Parental Education^a^	–	–	18.70	<.001	–	–	–	–	–	18.52	<.001	–	–	–
*Vocational*	−.14	.34	.18	.67	.87	.44	1.68	−.14	.34	.17	.68	.87	.45	1.69
*O-Level*	.04	.25	.03	.87	1.04	.62	1.70	.03	.25	.02	.90	1.03	.63	1.69
*A-Level*	.51	.25	4.16	.04	1.66	1.02	2.71	.50	.23	4.02	.045	1.65	1.01	2.68
*Degree*	.74	.29	6.27	.01	2.01	1.17	3.71	.73	.29	6.22	.01	2.08	1.17	3.70
Chronic Pain^†^								−.32	.15	4.84	.03	0.73	0.55	0.97
−2 Log-likelihood						1,739.20							1,734.47	
Hosmer and Lemeshow Test						9.48							9.23	
Sig.^b^						0.30							0.32	
Nagelkerke *R*^2^						0.234							0.237	

*Note.*

† Dichotomous variable categories: Sex=male/female; Chronic pain=yes/no.

a Parental Education reference category for comparison groups=Certificate of Secondary Education (CSE).

b A poor fit is indicated for a model if the significance value for the Hosmer and Lemeshow Test is less than 0.05.

When chronic pain was added in model 2, the total explained variance of Nagelkerke *R*^2^ values rose slightly from 23.4% to 23.7%, indicating that the predictive power of chronic pain was marginal for reporting a pathway to higher education. Finally, further analysis of predictor variables indicated that adolescents who reported no or non-chronic pain were 1.27 times more likely to report a pathway to higher education than adolescents with chronic pain, while girls were 1.39 times more likely than boys.

Identical hierarchical linear regression models were conducted with the outcome variable of educational qualification scores, however, no predictive power was found. See Supplementary Materials for details.

### Aim 3a. Pain characteristics and academic achievement measures

*T*-tests were undertaken within the chronic pain group to explore in greater depth how specific characteristics of their pain may relate to academic achievement measures. These found different levels of pain disability (*t*[595] = 1.74, *p* = .02, *d* = .17; *M*_Yes_ = 3.21, *SD*_Yes_ = 2.17, *M*_No_ = 3.60, *SD*_No_ = 2.51) and pain anxiety (*t*[601] = 2.55, *p* = .03, *d* = .25; *M*_Yes_ = 12.73, *SD*_Yes_ = 4.93, *M*_No_ = 14.03, *SD*_No_ = 5.80) between those who reported a pathway to higher education and those who did not, although weaker evidence suggested no differences in average pain intensity levels were observed (*t*[598] = 5.20, *p* = .38, *d* = .52; *M*_Yes_ = 4.58, *SD*_Yes_ = 1.95, *M*_No_ = 5.61, *SD*_No_ = 2.16). Those who reported a pathway to higher education within the chronic pain group reported fewer somatic symptoms than those who did not, (*t*[551] = 3.62, *p*<.001, *d* = .38; *M*_Yes_ = .96, *SD*_Yes_ = 1.37, *M*_no_ = 1.51, *SD*_no_ = 1.81). Equivalent analyses were also undertaken with the educational qualifications variable, however, no group differences were observed.

### Aim 3b. Single mediator models exploring the interaction between chronic pain and academic achievement

Regression and mediation analyses were next conducted to explore how difficulties with sleep, concentration, or fatigue may mediate the relationship between chronic pain and the measures of academic achievement (see Table 5). Across the regression and mediation analyses conducted, the path *a* from chronic pain to the mediator was significant at a *p*<.001 level for each mediator, indicating that chronic pain was a predictor of difficulties with sleep, concentration, and fatigue. For path *b* from the mediators to the academic achievement measures however, only difficulties with sleep predicted less likelihood of reporting a pathway to higher education which, similarly to the initial multiple regression models, was not found for the linear regression with educational qualification scores. Explorations of the moderating effect of the chronic pain characteristics of pain intensity, anxiety, and disability for the significant mediation model with sleep difficulties found that none of the pain characteristics moderated the relationship between chronic pain and sleep.

**Table 5.** Regression of academic achievement measures on chronic pain with mediators.

				Direct effect	Indirect effect	Indirect effect BC 95% CI	
*Mediator*	*N*	Path *a B (se)*	Path *b B (se)*	Path *c′ B (se)*	Path *ab B (se)*	*Lower*	*Upper*
**Logistic Regression of Pathway to Higher Education on Chronic Pain**							
Sleep	2,040	.27 (.06)**	−.15 (.06)*	−.24 (.15)	−.04 (.02) ^†^	−.08	−.01
Concentration	2,040	.26 (.05)**	−.09 (.07)	−.26 (.15)	−.02 (.02)	−.07	.02
Fatigue	2,040	.45 (.08)**	−.06 (.05)	−.26 (.15)	−.03 (.02)	−.07	.01
**Linear Regression of Educational Qualifications on Chronic Pain**							
Sleep	2,020	.26 (.06)**	−.06 (.07)	−.19 (.17)	−.02 (.02)	−.06	.02
Concentration	2,020	.25 (.05)**	−.05 (.08)	−.13 (.16)	−.01 (.02)	−.03	.06
Fatigue	2,020	.47 (.07)**	−.07 (.05)	−.18 (.17)	−.04 (.03)	−.08	.01

*Note.*

* *p*=.01.

** *p*<.001.

† Significant effect as no zero between the lower and upper bounds of the CI.

Logistic and linear models were also conducted which included identified mediators sequentially, although no significant direct or indirect effects observed within any of the models (see Supplementary Material).

## Discussion

Using data from the ALSPAC, the current study compared a community-based sample of adolescents with chronic pain to their peers without chronic pain across a range of psychosocial and behavioral outcomes as well as academic achievement measures. Results from the first research aim indicated that adolescents with chronic pain were more likely to report a range of mental health difficulties, including issues with sleep, concentration, and fatigue. They also more frequently reported instances of being bullied and reported use of tobacco and other substances more than their peers. For the second aim of this study, exploring group differences in academic achievement measures after confounding variables were accounted for, chronic pain negatively impacted the likelihood of obtaining qualifications associated with pursuing higher education, in what appears to be the first study of its kind among a U.K. population of adolescents. Results from Aims 3a and 3b reflect that adolescents with chronic pain who reported a pathway to higher education experienced fewer somatic symptoms (as compared to adolescents with chronic pain who did not pursue higher education) and that sleep appeared to have a modest, indirect effect on observed relations between chronic pain and academic achievement.

As found across other studies, adolescents with chronic pain were more likely to report the presence of a range of mental health difficulties relating to both anxiety and depression (Noel et al., 2016; Wager et al., 2020) as well as specific difficulties with fatigue (Wakefield et al., 2022), concentration, and sleep (Solberg Nes et al., 2009). Given the overall impact of these on daily functioning, there are important considerations for the role that depressive symptoms play in school impairment, such as absences and perception of academic competence (Logan et al., 2009), whereby it is necessary to further explore targeting such factors when addressing school functioning and academic achievement among adolescents with chronic pain.

Higher instances of smoking behaviors and cannabis use among adolescents with chronic pain are in line with some previous findings (McLaren et al., 2017; Zvolensky et al., 2010). Substance use and chronic pain have been theorized to positively reinforce one another in a manner which compounds and perpetuates both conditions in the context of self-medication to deal with aspects of chronic pain, such as its intensity, interference, and the fear of pain (Ditre et al., 2019). Although it was not possible to infer such causality within this study, results suggest further inquiry is warranted of the relationship between chronic pain and substance use in adolescents, particularly among community samples of adolescents who may not be accessing evidence-based pain treatment. Similarly, higher bullying rates among the chronic pain group were in line with the heightened risk of peer victimization discussed in the research literature (Fales et al., 2018); however, it was not possible for this study to explore known moderating and confounding factors that may influence this relationship, such as the level of support from peers or family (Marin et al., 2021).

The results of this study exploring the relationship between chronic pain and academic achievement are in line with the somewhat mixed findings within the research literature which have discussed both the absence of a relationship (Devanarayana et al., 2008) and the presence of a relationship between various measures of chronic pain and academic achievement (Turk & Şahin, 2020). The finding that those who reported a pathway to higher education experienced fewer somatic symptoms was also in line with previous research (Modin et al., 2015). Potential explanations of mixed findings may include differences in how chronic pain and academic achievement were operationalized, variances within a community chronic pain population, and the potential internal and external factors that may have enabled these adolescents to maintain a typical developmental trajectory at school despite experiencing chronic pain. Compared to their peers without chronic pain, young people with chronic pain have demonstrated superior problem-solving ability (Eccleston et al., 2008), communication skills, emotional maturity (Jordan et al., 2018), and the ability to perceive positive consequences when faced with adversity (Soltani et al., 2018). This complex coexistence of both delayed and accelerated areas of development is a critical consideration, therefore, in identifying the unique challenges that young people with chronic pain may face and the potential skills that they may possess to overcome them. Furthermore, Chomistek et al. (2019) observed that most students receive additional resources from teachers to support them with difficulties arising from chronic pain, while Vervoort et al. (2014) discussed how perceived teacher support of autonomy and school competence moderated the effect of chronic pain severity on perceived school performance.

Through the mediating role that sleep difficulties played, a modest, indirect effect was observed for chronic pain and reporting a pathway to higher education. This was in line with broader research on the impact of sleep on academic achievement (Dewald et al., 2010) and, indeed, the established prevalence of sleep difficulties among young people with chronic pain (Wager et al., 2020). Given the complex interrelationship between sleep, chronic pain, and subsequent difficulties in daily living, it is difficult to determine directionality of any effects (Finan et al., 2013), however, further investigation into the mediating role of sleep difficulties is required, including specific measures of daytime fatigue and the quality of sleep.

### Strengths and limitations

The secondary analysis utilized an existing cohort to access a large community sample population that would have traditionally been difficult to recruit due to high attrition rates and potential functional difficulties in participating in research (Jastrowski Mano et al., 2013). Several limitations are noted, however. As per cross-sectional analyses, the causal relationship between chronic pain, related factors, and measures of academic achievement cannot be established. Furthermore, as the relationship between sleep and chronic pain is complex and bidirectional, with overlapping negative outcomes for daily functioning (Finan et al., 2013), it is not possible to fully demarcate the way sleep mediates the relationship between chronic pain and the measures of academic achievement.

A critical point of note was that all significant findings among regressions and mediation analyses that related to academic achievement were found for the dichotomous pathway to higher education variable but not the continuous educational qualifications variable. This suggests that findings were less robust and were sensitive to changes in the level of measurement and operationalization of the outcome variable for academic achievement.

The ALSPAC’s assessment of pain history limited our ability to identify pain-related etiology and diagnoses. This restricts the generalizability of the findings due to its inability to distinguish between self-reported general experiences of chronic pain and specific conditions for which pain is the defining diagnostic feature, such as fibromyalgia or arthritis. As such, its relevance to specific clinical populations is limited. Additionally, no onset period of chronic pain was recorded, meaning that it was not possible to infer when any reported difficulties may have begun. The study also did not assess for pain-related treatments received, which may influence the trajectory of youth’s psychosocial and pain-related functioning. The chronic pain definition was, however, beneficial for incorporating a community population of adolescents with chronic pain, accounting for a potentially high proportion of those who experience chronic pain but do not report it to a clinician (Perquin et al., 2000).

Limitations of ALSPAC data include an overrepresentation of white, female, and middle-to-high-income families and high attrition rates (Boyd et al., 2013). As such, the generalizability of the results is limited for participants of other ethnicities or lower SES. Additionally, ethnicity data were collected using terminology that has changed since the data were collected, where best practice recommends naming specific ethnic groups rather than a collective reference such as “non-white.” It is also acknowledged that some relevant confounders with academic achievement were not included within the analyses of this study, such as lone-caregiver status or child maltreatment (Romano et al., 2015). This may have also been partially accounted for, however, by the confounding variables of SES and the highest level of caregiver education being strongly associated with such factors (Wright et al., 2018).

### Implications for practitioners

This study has outlined some of the unique difficulties a community-based population of adolescents with chronic pain experience and, potentially when no differences in academic achievement were observed, the skills they may have to overcome them (Eccleston et al., 2008; Jordan et al., 2018). To reach adolescents with chronic pain who may or may not be receiving pediatric pain care, a social–ecological approach is required in implementing strategies at community and institutional levels where adolescents would have access (McLeroy et al., 1988). This includes leveraging the important role that schools can play as a stable, structured, safe environment that facilitates the building of relationships and other resilience factors alongside capacities for learning (Nagel, 2009), which educational and clinical psychologists are well-placed to highlight. Similarly, this study’s findings of more frequent instances of sleep difficulties and their relationship with academic achievement has importance for psychologists, schools, and other health professionals working with this population. Community-based interventions for sleep difficulties highlighted by Rottapel et al. (2020) include providing education on sleep hygiene, developing positive sleep behaviors, and promoting stress management techniques. This can be achieved through building partnerships with schools, youth groups, social workers, local medical centers, or other relevant public services this population may frequent; whereby psychologists would be well-placed to inform and facilitate such work. Results of this study also suggest that schools and other professionals working with this population should be actively working to both prevent and treat substance use behaviors among this population, especially since adolescent chronic pain is an identified independent risk factor for opioid misuse in adulthood (Groenewald et al., 2019).

### Future directions

A more robust measure of chronic pain would benefit future studies, including an onset period and other data collection methods to self-report measures of pain, such as information on any diagnosed CP conditions or diagnostics undertaken by medical professionals as part of the data collection process, e.g., pain intensity and pain treatment. Future research should also feature more robust measures of academic achievement, such as a direct analysis of participants’ grades and their development over time, ideally measuring such grades before and after chronic pain is first experienced. It would additionally be beneficial to explore and stratify the findings of this study according to sex and age, the latter of which not being possible when data follow one cohort longitudinally. While this study identified sleep as an important factor for adolescents with chronic pain reporting a pathway to higher education, it was beyond its scope to explore the significance of depressive symptomology within this relationship or, indeed, other pertinent psychological and behavioral variables identified in the present study. Future research is, therefore, required to elucidate the directionality and strength of these relationships.

Further research should explore the relevant mechanisms present when no negative impacts on academic achievement or other school functioning are observed. This should also explore which interventions may be more beneficial for specific groups, such as based on gender or chronic pain condition (Day et al., 2015). Furthermore, most interventions for this group to date have been adapted from adult models, which risks inadequately targeting distinct features that apply to adolescents with chronic pain (Fisher et al., 2016). As such, a bottom-up framework is required which utilizes psychological, behavioral, and physiological responses to chronic pain that addresses the difficulties which may arise in a school context.

## Conclusion

Chronic pain and difficulties with sleep appear important considerations for the ability to report a pathway to higher education. Compared to their peers, adolescents with chronic pain were additionally found to experience more difficulties in a range of measures, including mental health, bullying, and substance use. Despite these, the resilience of this group has been established across the research literature, whereby a strengths-based approach can be applied to empower adolescents to thrive in a school despite experiencing chronic pain.

## Supplementary Material

jsaf015_Supplementary_Data

## Data Availability

The data that support the findings of this study are available from the ALSPAC study, but restrictions apply to the availability of these data, which were used under license for the current study, and so are not publicly available. The data are available subject to approval from the ALSPAC study Executive. The empirical dataset used in this study has been archived with the ALSPAC study under the project identifier B3834. For further information please see http://www.bristol.ac.uk/alspac/researchers/.

## Footnotes

1 14,451 pregnancies were enrolled in total, with 13,988 children surviving to 12 months. The cohort was augmented further when the oldest children were approximately 7 years of age, whereby eligible cases who did not join the initial study were included. This increased the total sample size to 15,454 pregnancies, of which 14,901 children survived to 12 months. Further information about the ALSPAC cohort can be found on the ALSPAC website (http://www.bristol.ac.uk/alspac/researchers/access/). Please note that the study website contains details of all the data that is available through a fully searchable data dictionary and variable search tool (http://www.bristol.ac.uk/alspac/researchers/our-data/). Linked health or educational records were not used for this study.
