# Supplementary material for: Exploring academic achievement and relevant risk factors among a community sample of adolescents with chronic pain compared to peers
Source: J Pediatr Psychol. 2025 Apr 12;50(6):467–78. doi: 10.1093/jpepsy/jsaf015 (PMC12206300; doi:10.1093/jpepsy/jsaf015)
Supplement: jsaf015_Supplementary_Data [file jsaf015_supplementary_data.zip › jsaf015_Supplementary_Data/jpepsy-2024-0016-File007.docx]

**Supplementary Information: Computing Academic Achievement Measures**

**Variables Used to Compute Dichotomous ‘Pathway to Higher Education’**

Survey responses were added to the computed variable sequentially in the order that they were presented below. As such, if a later response indicated that a young person (YP) who had initially been assigned to the ‘no’ or ‘yes’ group had subsequently reported such a pathway that required this to be changed, they would be assigned to the most up to date group

| Question | Response | Pathway to Higher Education Group |
| --- | --- | --- |
| YP plans to leave full time education and work full or part time  YP plans to leave full time education and learn a trade/start work based training  YP plans to leave full time education and look after the family and home  YP plans to leave full time education and do nothing  YP plans to leave full time education and do something else  YP plans to leave full time education and doesn't know what they will do  Likelihood that the YP will go to university within the next 5 years  Respondent obtained qualification: GCSE grades A*-C  Respondent obtained qualification: GCSE grades D-G  Respondent obtained qualification: A/AS levels  Respondent obtained qualification: A/A2s  Respondent obtained qualification: AVCEs (formerly vocational A levels)  Respondent's main educational or training activity at the moment  Main reason respondent left full time education = no if any answer indicated they have left full time education / did not have pathway to higher education  Respondent applied to go to university  Number of choices of university respondent put on UCAS form  Respondent awarded place at university  Respondent got first choice of university  Respondent got current university through clearing  Has degree-level qualification including foundation degrees, graduate member  Has HNC/HND qualification  Has A-level/Vocational A-level/GCE in applied subjects or equivalents qualification | “Yes”  “Yes”  “Yes”  “Yes”  “Yes”  “Yes”  “Not at all likely”  “Yes”  “Yes”  “Yes”  “Yes”  “Yes”  “Not engaged in education or training”  Any reason for leaving full-time education  “Yes” or “No  “Not applied”  “Yes”  “Yes”  “Yes”  “Yes”  “Yes”  “Yes” | No  No  No  No  No  No  No  Yes  Yes  Yes  Yes  Yes  No  No  Yes or No  No  Yes  Yes  Yes  Yes  Yes  Yes |

**Variables Used to Compute Continuous ‘Educational Qualifications’**

| **Academic Achievement Measure** | **Score Assigned** |
| --- | --- |
| Obtained GCSE grade D-G  Obtained GCSE grade A*-C  Obtained A/AS levels  Obtained A/A2s levels  Obtained AVCEs  Obtained ONC/OND  Obtained HNC/HND  Has Degree (incl. foundation degree, grad. membership, PGCE, or higher) | 0  1  1  2  3  3  3  4 |
