# Supplementary material for: Exploring academic achievement and relevant risk factors among a community sample of adolescents with chronic pain compared to peers
Source: J Pediatr Psychol. 2025 Apr 12;50(6):467–78. doi: 10.1093/jpepsy/jsaf015 (PMC12206300; doi:10.1093/jpepsy/jsaf015)
Supplement: jsaf015_Supplementary_Data [file jsaf015_supplementary_data.zip › jsaf015_Supplementary_Data/jpepsy-2024-0016-File008.docx]

**Supplementary Information: Sequential Mediation Models**

To further investigate the complex inter-relationship between CP, sleep problems, and subsequent difficulties with concentration and fatigue, logistic and linear regression models were run which included mediators sequentially, with sleep as M_1_ and concentration or fatigue as M_2_ (Figure S1). This explored whether concentration and fatigue difficulties, which may arise from impacted sleep, were significant contributing factor in the mediating role that sleep plays in the relationship between CP and measures of academic achievement.

**Figure S1**

|  |
| --- |

*Regression Models with Sequential Mediators: Sleep, Concentration, Fatigue*

*Predictor X:* Chronic Pain

*Outcome Y:* Pathway to Higher Education, Educational Attainment

*c’*

*b_2_*

*a_1_*

*Covariates:* Sex, IQ, SES, Parental Education

*M_1_*  Sleep

*M_2_*  Concentration, Fatigue

*d_1_*

*a_2_*

*b_1_*

As outlined in Tables S1 and S2, no direct or indirect effects were observed in either logistic or linear regression models, indicating that our measures of concentration or fatigue do not offer explanatory power in the role that sleep plays in mediating the relationship between CP and reported pathway to higher education.

**Table S1**

*Logistic Regression Models with the Mediators of Sleep and Subsequent Difficulties.*

| *Direct Effect* |  |  | *Indirect Effect* | *BC 95% CI* | |
| --- | --- | --- | --- | --- | --- |
| Path *c’*  B (se) | *p* | *Relationship* | Path *a_1_d_1_b_2_*  B (se) | *Lower* | *Upper* |
| -.23 (.15) | .13 | *Chronic Pain → Sleep → Concentration → Pathway to Higher Education (n = 2040)* | -.002 (.00) | -.01 | .01 |
| -.24 (.15) | .13 | *Chronic Pain → Sleep → Fatigue → Pathway to Higher Education*  *(n = 2040)* | -.003 (.01) | -.02 | .01 |

**Table S2**

*Linear Regression Models with the Mediators of Sleep and Subsequent Difficulties.*

| *Direct Effect* |  |  | *Indirect Effect* | *BC 95% CI* | |
| --- | --- | --- | --- | --- | --- |
| Path *c’*  B (se) | *p* | *Relationship* | Path *a_1_d_1_b_2_*  B (se) | *Lower* | *Upper* |
| -.21 (.17) | .23 | *Chronic Pain → Sleep → Concentration → Educational Qualifications (n = 2020)* | .004 (.01) | -.01 | .02 |
| -.17 (.17) | .31 | *Chronic Pain → Sleep → Fatigue → Educational Qualifications*  *(n = 2020)* | -.01 (.01) | -.02 | .01 |
