# Supplementary material for: Exploring academic achievement and relevant risk factors among a community sample of adolescents with chronic pain compared to peers
Source: J Pediatr Psychol. 2025 Apr 12;50(6):467–78. doi: 10.1093/jpepsy/jsaf015 (PMC12206300; doi:10.1093/jpepsy/jsaf015)
Supplement: jsaf015_Supplementary_Data [file jsaf015_supplementary_data.zip › jsaf015_Supplementary_Data/jpepsy-2024-0016-File009.docx]

**Supplementary Information: Linear Regression Models with ‘Educational Qualifications’ Outcome Variable**

A hierarchical linear regression model introduced the covariates of sex, IQ, SES, and parental education in step 1 (*F*[4, 2188] = 50.48, *p* = <.001, *R^2^_adjusted_* = .083) of the analysis before adding CP in step 2 (*F*[5, 2187] = 40.52, *p* = <.001, *R^2^_adjusted_* = .083) in assessing their predictive value for continuous educational qualification scores, with low R^2^ values indicating a poor fit for both models and minimal change when CP was added. While both models predicted 8.3% overall of the variance of the outcomes variable, CP did not predict educational qualification scores when it was included in model 2 (*B* = -.02, *p* = .40), with the covariates accounting for most of the predictive value of this model (Sex: *B* = .12, *p* < .001; IQ: *B* = .18, *p* <.001; SES: *B* = .08, *p* = .001; parental education: *B* = .09, *p* = <.001).
